# Derivation-Based Calibration of IMUs Using Savitzky–Golay Filters

**DOI:** 10.3390/s26061788

**Published:** 2026-03-12

**Authors:** Diogo Vieira, Miguel Oliveira, Rafael Arrais

**Affiliations:** 1 Department of Mechanical Engineering, University of Aveiro, 3810-193 Aveiro, Portugal; mriem@ua.pt; 2 Institute of Electronics and Informatics Engineering of Aveiro (IEETA), University of Aveiro, 3810-193 Aveiro, Portugal; 3 Intelligent Systems Associate Laboratory (LASI), School of Engineering, University of Minho, 4800-058 Guimarães, Portugal; 4 Institute for Systems and Computer Engineering, Technology and Science (INESC TEC), 4200-465 Porto, Portugal; rafael.l.arrais@inesctec.pt; 5 Faculty of Engineering, University of Porto, Rua Dr. Roberto Frias, 4200-465 Porto, Portugal

**Keywords:** robotic system calibration, inertial measurement unit, extrinsic calibration, Savitzky–Golay filter

## Abstract

For any robotic application, accurate sensor calibration is crucial. In the case of mobile platforms or flying drones, a sensor commonly utilized is the Inertial Measurement Unit (IMU). Current approaches to the calibration of IMU-equipped robotic systems focus on sensor-to-sensor calibration, meaning a second sensor is necessary for the calibration process. Furthermore, a great number of those rely on integrating the sensor measurements to obtain its pose, which leads to integration errors. In this work, we present a method for the extrinsic calibration of IMUs in robotic systems, which avoids the errors originating from IMU integration by instead taking a derivative approach using Savitzky–Golay filters. The proposed calibration method estimates the transformation between an IMU sensor and its parent frame in the system’s kinematic chain by minimizing the differences between derived linear accelerations and angular velocities and those measured by the sensor. Simulated data is used to establish a ground truth against which the calibration results are compared. Results indicate that the proposed method achieves a higher accuracy than the alternatives it is compared against, while also showing the method can be applied to industrial-grade IMUs.

## 1. Introduction

IMUs are sensors, typically comprised of an accelerometer and a gyroscope, which collect data regarding the dynamics of a given body. They are commonly used in Inertial Navigation Systems (INS), which are present in a variety of applications, such as mobile platforms and flying drones, where their data is often used for stabilization [1]. Other IMU applications include Simultaneous Localization and Mapping (SLAM) [2], 3D reconstruction [3], light sensing systems for pipeline defect inspection [4] and wearable exoskeleton robots [5]. Due to the presence of IMUs in a wide variety of systems, the calibration of IMU-equipped systems is an important issue that warrants exploration.

Calibration is a fundamental procedure for the proper usage of any robotic system, from those containing a stereo optical setup [6,7] to those with multi-modal sensor pairings [8]. One aspect of calibration is intrinsic calibration. Intrinsic calibration aims to estimate the intrinsic parameters of the sensor model. On the other hand, the extrinsic calibration of a sensor is the process of determining the position and orientation of that sensor in a system. For systems using data from an IMU, it is important to accurately determine the pose of the IMU in relation to the rest of the system.

Calibration processes can take two forms. The first is offline calibration, where the calibration is performed after a data collection process. The second is online calibration, where the calibration is a continuous process that occurs alongside sensor operation. Current research efforts concerning the calibration of IMU-equipped systems focus mostly on online calibration methods [8,9,10]. Although offline calibration methods have also been explored in the literature [11,12,13], we have found none that does not depend on the presence of a second sensor, with a time-invariant spatial relation to the IMU.

Approaches to extrinsic IMU calibration found in the state of the art operate on a sensor-to-sensor paradigm [11,12,13], meaning the transformation between the IMU and a second sensor is estimated. This is a limitation of the methods presented thus far, as they only allow for the estimation of an IMU pose if there is a second sensor in the system and if that sensor has a rigid transformation w.r.t. the IMU.

In summary, the contributions in this paper are:

- A derivation-based method for the extrinsic calibration of IMUs in robotic systems, which makes use of Savitzky–Golay filters [14] for the purposes of motion data derivation and does not require the presence of other sensors;
- An assessment of the impact of noise in the IMU sensor data on the accuracy of the proposed calibration method;
- An assessment of the sensitivity of the proposed method to the initial error in the estimated IMU sensor extrinsics.

The structure of this work is as follows: the existing literature on the calibration of IMU-equipped robotic systems is laid out in Section 2. In Section 3, the proposed approach to the derivation of the motion data and the cost function formulation is laid out. Additionally, the test cases used to validate our approach are also presented. In Section 4, the results of our tests are presented, interpreted and compared to the state of the art. Conclusions are drawn in Section 5.

## 2. Related Work

As mentioned, methods for the calibration of IMU-equipped systems found in the state of the art operate on a sensor-to-sensor paradigm. Publications on the topic revolve around the calibration of IMU–camera sensor pairs [12,15,16,17,18,19,20,21], IMU–LiDAR sensor pairs [11], and IMU–camera–LiDAR configurations [8].

In 2008, the camera–IMU calibration problem was tackled using Kalman filters [17], while also considering the time correlation between IMU measurements. The method proposed by Mirzaei and Roumeliotis [17] uses the fourth-order Runge–Kutta (RK4) numerical integration method whenever a new IMU signal is sampled to determine the IMU orientation and position w.r.t. a global frame. This data is then used to describe the state of the system in the Kalman filter.

A method proposed by Furgale et al. [15] combines spatial and temporal calibration. This is done by simultaneously estimating the temporal offset between camera and IMU measurements as well as the the camera–IMU transformation. The Levenberg–Marquardt (LM) algorithm is used in the optimization process. This method then becomes the foundation of the Kalibr calibration toolbox (https://github.com/ethz-asl/kalibr (accessed on 15 August 2025)). In 2017, Rehder and Siegwart [20] built on this work, taking into account the displacement of individual accelerometers, pointing this out as a source of error for IMU with several single-axis accelerometers, for example.

The classic formulation of hand–eye calibration, which estimates the transformation between a manipulator’s end-effector and a camera sensor mounted on it, has been adapted for camera–IMU calibration. Liang et al. [16] use this formulation of the problem. Using the Perspective-n-Point (PnP) algorithm [22], they compute the pose of the camera relative to a calibration pattern. Doing this at two distinct times, the authors were able to compute the displacement. On the other hand, the authors also integrated the IMU data to obtain that same displacement. The difference between the two displacements is then used to evaluate the accuracy of the estimated camera–IMU transformation. A Random Sample Consensus (RANSAC) process is used to eliminate outliers, using that difference as the criteria.

Wu et al. [19] propose a calibration method that simultaneously performs hand–eye and camera–IMU calibration. The IMU data is not integrated in order to avoid errors inherent to the integration process. The method proposed in this paper is similar in this regard, as it also forgoes IMU data integration. However, the method proposed by Wu et al. [19] operates on a sensor-to-sensor paradigm, which differs from the method detailed in Section 3 of this paper.

Ouyang et al. [21] point out a key problem in camera–IMU calibration: to achieve enough excitation of the IMU and obtain enough information for calibration, camera data acquisition can be more difficult due to the motion. The authors resolve this issue by introducing a motion capture system to their robotic system. The authors are then able to extrinsically calibrate the motion capture system and the IMU, as well as the motion capture system and the camera, via continuous-time trajectory estimation. From this, the authors can compute the IMU–camera extrinsic parameters.

Other notable works include those of Wang et al. [8], Chi et al. [18], and Yin et al. [11]. Chi et al. [18] proposed a method for the calibration of underwater systems with an IMU and multiple cameras using the LM algorithm for parameter optimization. Their method features the integration of IMU data to generate a stream of IMU poses, which are then interpolated using B-spline curves [23] to align temporally with camera data. Wang et al. [8] presented a method for the online calibration of IMU–camera–LiDAR setups where the IMU serves as a bridge between camera and LiDAR. The IMU–camera and IMU–LiDAR transformations are separately estimated, and the camera–LiDAR transformation is derived from them afterwards. This method also makes use of IMU data integration during IMU–camera calibration. Yin et al. [11] integrated IMU data over time in their method for IMU-LiDAR rotation calibration.

The works presented in this section assume there is a static transformation between the IMU sensor and a second sensor, such as a camera. In contrast, the method proposed in this work estimates the transformation between an IMU sensor and a generic frame, such as the end-effector of a manipulator, without the need for a second sensor. This allows for application in cases where a second sensor is not present.

## 3. Proposed Approach

**Figure 1:**
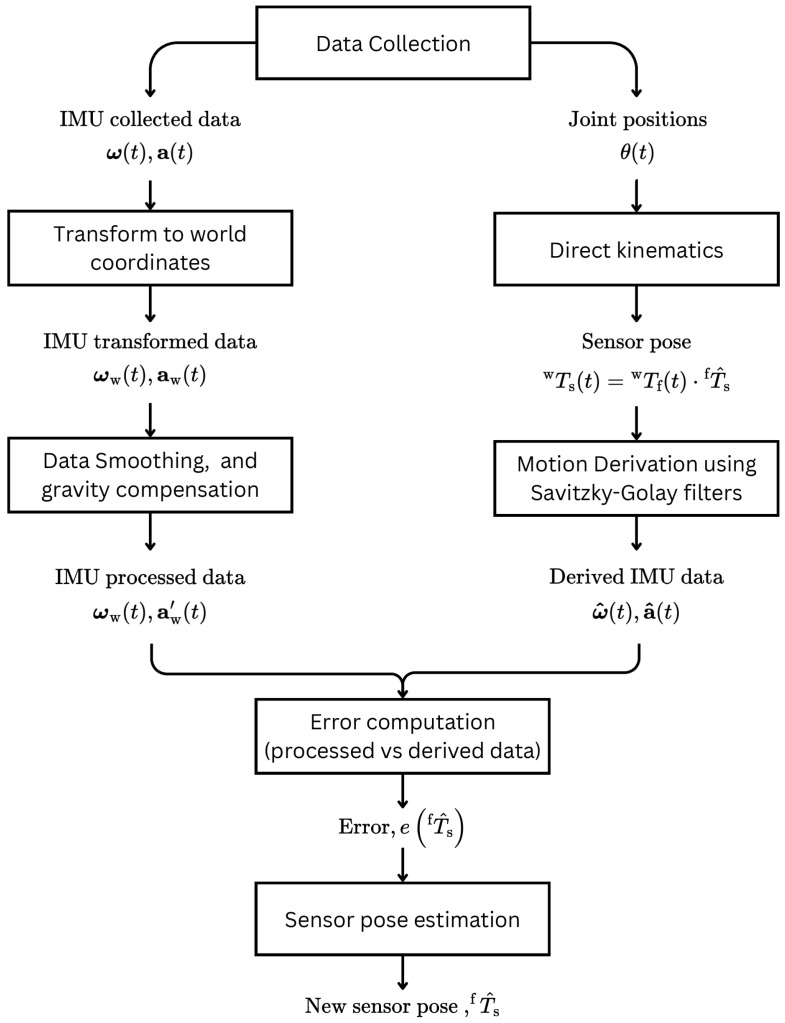
A block diagram describing the proposed IMU calibration method.

We propose a novel method for the calibration of IMU-equipped systems. A diagram of the proposed method can be found in Figure 1. Unlike current methods, this calibration does not estimate the transformation between an IMU and a second sensor, but instead the transformation between an IMU and a reference frame in the kinematic chain of the robotic system. As such, this method allows for the calibration of IMU extrinsics regardless of whether there is a second sensor involved. As a corollary to this, calibration is also possible when the transformation between the IMU and a second sensor is dynamic. An example of this is a system composed of a robotic manipulator with an IMU attached to it and a separate static vision module.

The calibration process itself revolves around a nonlinear least squares optimization. The proposed formulation of the cost function avoids the typical trappings of methods which involve integrating the IMU data over time, by taking the reverse approach of data derivation. The method employed for the derivation of the motion data is presented in Section 3.1, while the cost function formulation is explored in Section 3.2.

### 3.1. Motion Data Derivation

It is worth noting the coordinate frames relevant to the proposed method. These are the world frame, denoted by “w”; the parent frame of the IMU sensor frame, which precedes it in the kinematic chain of the robotic system, “f”; and finally, the IMU sensor frame, denoted by “s”.

The proposed approach for calibration requires a dataset containing the transformations from a global frame to the parent frame of the IMU sensor frame, Tfw(t), computed from the joint positions of the robotic system, θ(t), as well as the IMU data collected during that time, namely its acceleration $a_{\mathrm{IMU}}\left(t\right)$ and its angular velocity $\omega_{\mathrm{IMU}}\left(t\right)$.

Through direct kinematics, the transformation from the global frame to the IMU sensor frame at each instant *t*, Tsw(t) can be calculated:

(1) Tsw(t)=Tfw(t)·Tsf

where Tsf is the time-invariant transformation from the parent frame of the IMU sensor frame to the IMU sensor frame. In fact, it is this transformation which is calibrated during the optimization process.

Tsw(t) can be decomposed into a translation vector τ(t) and a 3 × 3 rotation matrix R(t). These can be directly extracted from the homogenous transformation matrix. τ(t) is composed of the first three values in the fourth column of Tsw(t), while R(t) is composed of the first three rows and three columns of the transformation matrix. From τ(t), through derivation w.r.t. *t*, we have:

(2) $$a\left(t\right)=\frac{d^{2}}{dt^{2}}\tau \left(t\right)$$

where a(t) is the linear acceleration of the IMU sensor frame from the perspective of the world frame. Similarly, we can also obtain the first temporal derivative of R(t), $\dot{R}\left(t\right)$. Both a(t) and $\dot{R}\left(t\right)$ can be obtained using Savitzky–Golay filters.

Savitzky–Golay filters can be used to both smooth and derive data series through convolution [14]. The filter fits subsets of what is, in this case, a time series, to a polynomial function. Consider a set of *N* measurements of the world–IMU translation, $\tau_{x_{j}},j\in \left\{1,\ldots ,N\right\}$, and a filter window length of *L* measurements. The filter is applied to each measurement as such:

(3) $$\tau_{x_{j}}^{★}=\sum_{k=-\frac{L-1}{2}}^{\frac{L-1}{2}}C_{k}\tau_{x_{j+k}}$$

where $\tau_{x_{j}}^{★}$ is the filtered value relative to the *j*-th measurement of the world-IMU translation along the X axis; $C_{k}$ is the *k*-th value in a set of *L* convolution coefficients. Note that, depending on the coefficient *C* used, $\tau_{x_{j}}^{★}$ can represent a smoothed value for the translation $\tau_{x_{j}}$ or for its derivatives.

For the case at hand, a window length L=5 was used. A small window length is used to allow for the Savitzky–Golay filter to match rapidly changing measurements. The convolution coefficients used in the filter correspond to a third degree polynomial function in order to avoid overfitting. This is especially relevant since one of the purposes behind the use of the Savitzky–Golay filter is to mitigate the effect of noise in the sensor measurements. Furthermore, proper fitting requires the order of the polynomial function to be less than the number of measurements used.

The transformation data gathered in the dataset has a constant frequency. The convolution coefficients for this fitting, for cases with a constant interval between measurements, have been determined and can be applied. In this case, Savitzky–Golay filters are applied to each element of both the τ(t) vector and the R(t) matrix, with the corresponding convolution coefficients, in order to compute the second derivative of the translation data and the first derivative of the rotation data. In Section 4, graphical representations of smoothed translation data can be found, along with its first and second derivatives.

With $\dot{R}\left(t\right)$ and the transpose of the rotation matrix, $R^{T}\left(t\right)$, it is possible to get the angular velocity matrix Ω(t) from which we can extract the angular velocity vector $\omega \left(t\right)=\left(\omega_{x}\left(t\right),\omega_{y}\left(t\right),\omega_{z}\left(t\right)\right)$ [24]:

(4) $$\Omega \left(t\right)=\dot{R}\left(t\right)\times R^{T}\left(t\right)$$

From this, we can obtain ω(t):

(5) $$\Omega \left(t\right)=\left[\begin{array}{ccc} 0 & -\omega_{z}\left(t\right) & \omega_{y}\left(t\right)\\ \omega_{z}\left(t\right) & 0 & -\omega_{x}\left(t\right)\\ -\omega_{y}\left(t\right) & \omega_{x}\left(t\right) & 0 \end{array}\right]$$

As such, we can calculate the linear acceleration and angular velocity of the IMU sensor frame w.r.t. the world frame.

### 3.2. Cost Function Definition

Consider a generic vector q, containing linear accelerations and angular velocities, such that $q=\left(a_{x},a_{y},a_{z},\omega_{x},\omega_{y},\omega_{z}\right)$. Assume the transformation between the IMU parent frame and the sensor is perfectly estimated, i.e., T^sf=Tsf, and that the IMU measurements are also perfect. Under these circumstances, we should have, for any given instant:

(6) $$q_{\mathrm{derived}}=q_{\mathrm{measured}}^{\prime }$$

where $q_{\mathrm{derived}}$ is the vector obtained by the derivation process and $q_{\mathrm{measured}}^{\prime }$ is the corresponding IMU measurements vector, expressed in the world frame and unaffected by gravity. From the acceleration and angular velocity vectors sampled by the IMU, which are expressed in its local frame, we can compute:

(7) $$a_{w}=\hat{R}a_{\mathrm{IMU}}$$

(8) $$\omega_{w}=\hat{R}\omega_{\mathrm{IMU}}$$

where $\hat{R}$ is the estimated 3×3 rotation matrix from the world frame to the IMU frame, extracted from T^sw. Note that this matrix consists of the first three rows and first three columns of the transformation matrix.

To compute $q_{\mathrm{measured}}^{\prime }$ and in order for the measured and derived acceleration to be properly compared, gravity must be compensated for. The gravitational acceleration, g, is measured by an IMU as a vertical vector pointing upward. In the world frame, this translates to an acceleration vector with a positive value in the Z axis:

(9) $$g_{w}=\left(0\;0\;g\right)$$

As such, we can compensate for gravity:

(10) $$a_{w}^{\prime }=a_{w}-g$$

(11) $$q_{\mathrm{measured}}^{\prime }=\left(a_{w}^{\prime },\omega_{w}\right)$$

With this, a comparison can be established between the expected IMU measurements, acquired through the derivation of the world–IMU transformation and the IMU measurements collected from the sensor readings and expressed in the world frame. It is worth noting that the IMU measurements are also subjected to a Savitzky–Golay filter in order to smooth the IMU signals and minimize the impact of noise in the sensor readings. This comparison is the basis for the proposed error function to be optimized. The error is defined as the L1 distance or Manhattan distance between the derived vector $q_{\mathrm{derived}}$ and the measured and gravity-compensated vector $q_{\mathrm{measured}}^{\prime }$, formulated in relation to an estimate of the transformation between the IMU sensor frame and its parent frame, T^sf:

(12) eT^sf=qderivedT^sf−qmeasured′1=∑k=16qkderivedT^sf−qkmeasured′

where $q_{k}$ is the k-th element of the vector q.

In reality, that error corresponds to a single instant. Consider a set of N measurements, each taken at a time instant $t_{i},\;i\in \left\{1,2,\ldots ,N\right\}$. The optimization can then be formulated as the minimization of the sum of each error term:

(13) arg minT^sf∑i=1Nqderivedti,T^sf−qmeasured′(ti)1

## 4. Results

In this section, the results of the proposed calibration method will be presented. Firstly, in Section 4.1, the method for testing the validity and accuracy of the proposed method is presented, as well as the simulated robotic systems used. In Section 4.2 the efficacy of the motion data derivation will be shown graphically. In Section 4.3, the results achieved in the calibration of RiHIBot are compared with those achieved using other methods from the literature. In Section 4.4, results of the calibration for both systems are presented, with varying levels of noise in the IMU readings. Finally, the sensitivity of the calibration method to the initial error in the transformation estimate is explored in Section 4.5.

### 4.1. Test Cases

In order to test this calibration method, several experiments were conducted. Simulated systems were used in order to compare the calibration results against a ground truth, directly obtained from the simulation.

**Figure 2:**
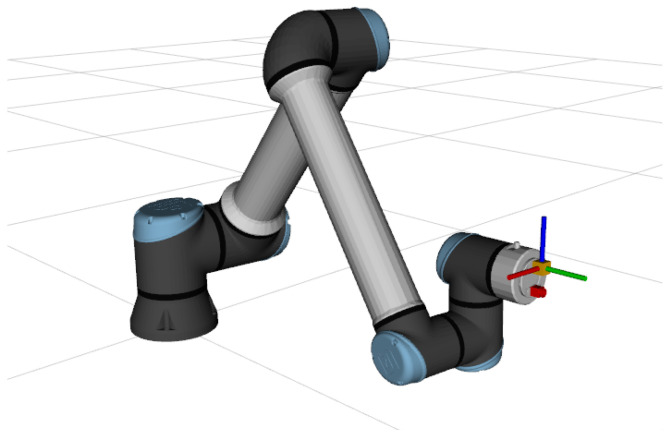
The RiHIBot system contains an UR10e robotic manipulator, with an IMU and an RGB camera attached to its end-effector. The IMU is represented in orange and the camera in red. Contrary to BarBot, this system is affected by gravity.

The first system used in testing is BarBot, a simplistic simulated system consisting of a floating bar with a mounted IMU sensor, operating at a frequency of 150 Hz. This simulated system is not affected by gravity. The second system, named RiHIBot, is comprised of an UR10e manipulator, equipped with an IMU and an RGB camera attached to its end-effector. The IMU used in this system also operates at a frequency of 150 Hz. This system is affected by gravity, with $g=9.81\;m\;s^{-2}$. An image of RiHIBot can be found in Figure 2. The Webots simulator was used, as well as the Open Dynamics Engine (https://www.ode.org/) dynamic model.

On the one hand, performing the calibration of the simulated BarBot allows the assertion of the viability of this calibration method in an environment where no other sensors are present. On the other hand, the RiHIBot system allows for the comparison to other calibration methods, which necessitate the presence of a camera. Furthermore, from the comparison of the results for both systems, the efficacy of the gravity compensation method employed by the proposed method can be observed.

**Figure 3:**
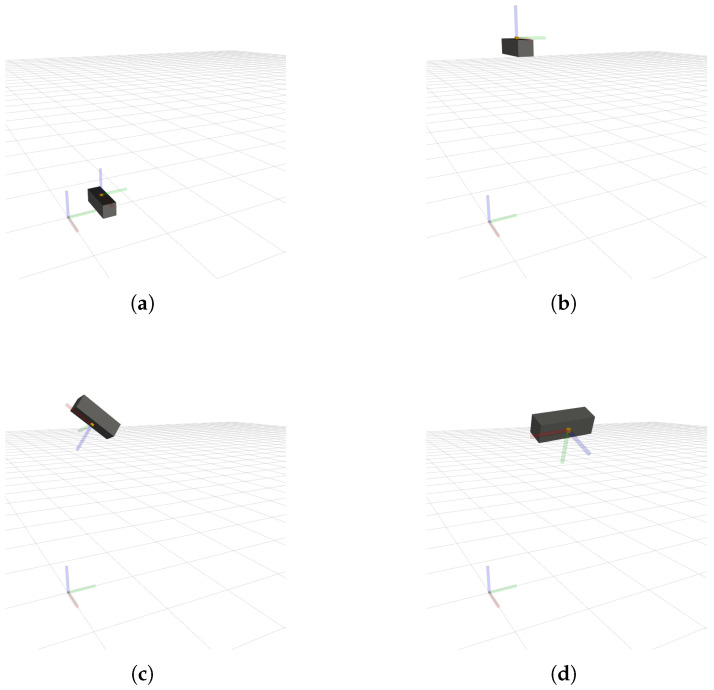
Four different poses of BarBot during data collection for calibration: (**a**) initial pose; (**b**) pose after upwards translation along the Z axis; (**c**,**d**) poses captured during a rotation motion. On the grid, the world coordinate system can be seen, while the coordinate system on BarBot corresponds to the IMU sensor frame.

**Figure 4:**
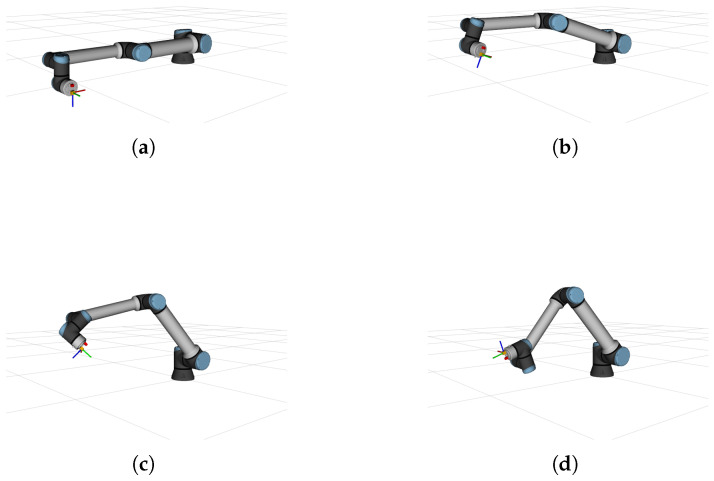
Four different poses of RiHIBot during data collection for calibration: (**a**) initial pose; (**b**) second pose; (**c**) third pose; (**d**) fourth pose. The coordinate system on RiHIBot corresponds to the IMU sensor frame.

Figure 3 shows different poses of the BarBot system during its simulation, while Figure 4 illustrates the motion of RiHIBot. The BarBot system simulation begins with a vertical movement upward, followed by a rotation around its three axes. Following this, a sequence of three translations along the global X, Y and Z axes are carried out in that order. Rotations around the X, Y, and Z axes are then also carried out in that order. The simulation has a duration of 128 s. The simulation for RiHIBot, on the other hand, comprises more complex movements, with several joints of the manipulator being actuated at once. The motion of the robot begins by positioning itself in front of a calibration pattern., followed by several repositioning motions in front of that pattern. The duration of the simulation is 74 s. It should be noted that the difference in duration between the two simulations is largely due to greater pauses between the various movements in the BarBot simulation. Furthermore, it should be noted that the calibration pattern in the simulation of the RiHIBot system is necessary for the comparisons with camera–IMU calibration methods in the literature (Section 4.3), but is not necessary for the IMU calibration method proposed in this work. As mentioned, the ground truth against which the calibration results are compared is directly obtainable from the simulation.

To test the sensitivity of our calibration method to noise in the IMU data, calibrations are carried out for each system with varying levels of noise in the accelerometer and gyroscope data sampled by the IMU. To do this, the IMU measurements are modeled as such:

(14) $$a=a_{\mathrm{measured}}+\lambda_{n,a}n_{a}$$

(15) $$\omega =\omega_{\mathrm{measured}}+\lambda_{n,\omega }n_{\omega }$$

with $\lambda_{n,a},\lambda_{n,\omega }\in R$ and $n_{a},n_{\omega },b_{a},b_{\omega }\in R^{3}$. The noise for the linear acceleration and angular velocity measurements is represented by $n_{a}$ and $n_{\omega }$, respectively. For each of these noise vectors, the elements are sampled from a Gaussian distribution:

(16) $$n_{\theta }∼N\left(1,0\right),\;\theta \in \left[x,y,z\right]$$

Additionally, the sensitivity of the calibration method to errors in the initial estimate of Tsf is also tested. To this end, an error vector with fixed norm and random direction is defined for both translation and rotation. Consider a pose Tsf, represented by a translation vector τ and a rotation vector r. The error-affected vectors are:

(17) $$\tau_{\mathrm{new}}=\tau +e_{\tau }\frac{\epsilon_{\tau }}{∥\epsilon_{\tau }∥}$$

(18) $$r_{\mathrm{new}}=r+e_{r}\frac{\epsilon_{r}}{∥\epsilon_{r}∥}$$

where $e_{\tau }$ and $e_{r}$ are the norms of the error term vectors for translation and rotation, respectively. $\epsilon_{\tau },\epsilon_{r}\in R^{3}$ are vectors composed of three randomly chosen values, which are divided by their norms to define the directions of the error term vectors.

### 4.2. Derivation Results

**Figure 5:**
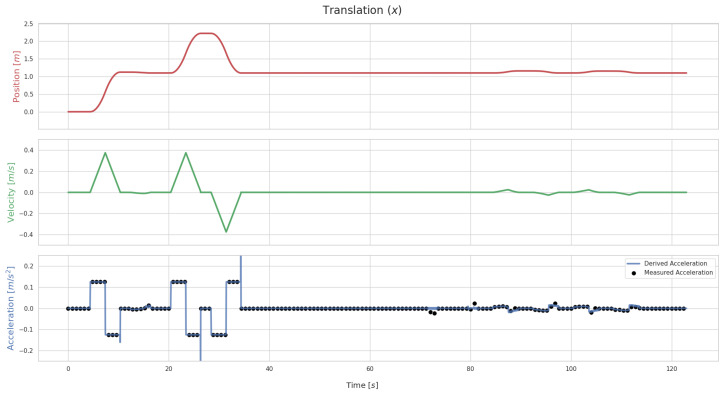
Translation values for the BarBot system along the X axis of Tsw when no error is applied to Tsf. The plot includes the position values, $\tau_{x}\left(t\right)$, and its first and second derivatives: the velocity and acceleration along X. In the accleration graph, two different values are plotted out. These are the acceleration from the derivation, through the use of Savitzky–Golay filters (blue line) and the acceleration measured by the IMU sensor (black dots).

**Figure 6:**
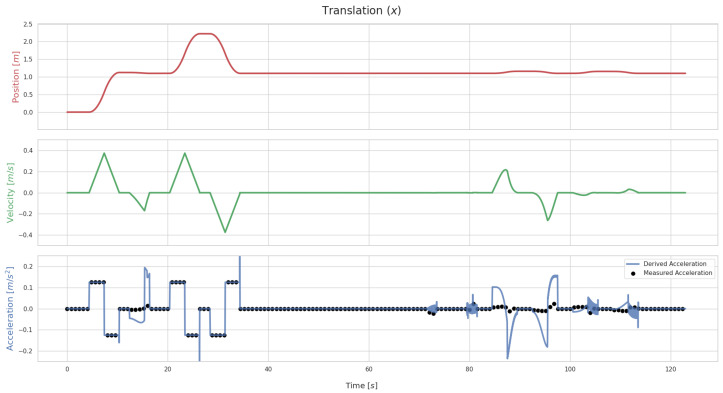
Translation values for the BarBot system along the X axis of Tsw when an error of 0.5 m in translation and 0.5 rad in rotation is applied to Tsf. The plot includes the position values, $\tau_{x}\left(t\right)$ and its first and second derivatives: the velocity and the acceleration along X. In the accleration graph, two different values are plotted out. These are the acceleration from the derivation, through the use of Savitzky–Golay filters (blue line), and the acceleration measured by the IMU sensor (black dots).

The proposed calibration method relies on the derivation of the world–IMU geometric transformation over time. Figure 5 plots out the values of the translation along the X axis of Tsw for BarBot, when no initial error is applied to the transformation. For the acceleration, the derived values are plotted alongside the IMU data. Conversely, Figure 6 shows the same values for when the initial estimate is affected by an error of 0.5 m in translation and 0.5 rad in rotation.

On the one hand, the results seen in Figure 5 indicate that the derivation method used is appropriate. This can be seen in how the acceleration derived from $\tau_{x}\left(t\right)$ matches almost perfectly with the acceleration data acquired from the IMU data stream. The exceptions to this are moments when movement along the X axis either starts or stops. To ensure the data used for optimization accurately represents the movement of the sensor, these moments where the derived acceleration brusquely varies are not included in the process.

On the other hand, Figure 6 illustrates how errors in the estimate of the transformation between the IMU and its parent frame impacts the derivation. A clear mismatch is visible whenever there is variation of position and velocity. This serves as an indication that convergence of the optimization process through the use of this mismatch between the derived values and those obtained from the sensor is possible.

**Figure 7:**
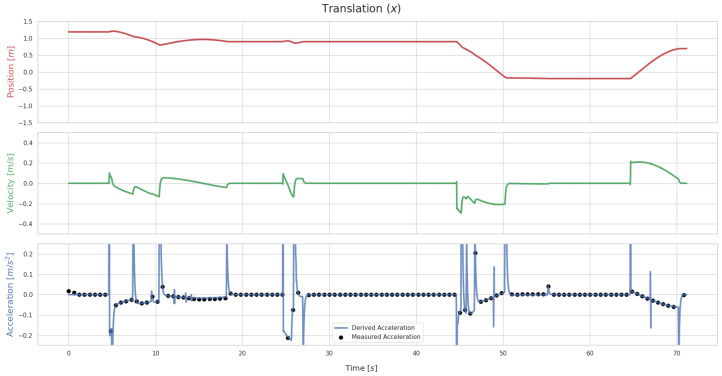
Translation values for the RiHIBot system along the X axis of Tsw when no error is applied to Tsf. This graph shows sporadic instances where there is a mismatch between the derived acceleration along the X axis and the measured acceleration.

Figure 7 shows the translation values along the X axis over time for the RiHIBot system, which is affected by gravity. A comparison of Figure 5 and Figure 7 shows that the addition of gravity to the simulated environment has minimal impact on the derivation process. This is also an indication that the method employed for the compensation of gravity in the IMU readings is adequate for the puposes of establishing a comparison between derived and measured acceleration values.

### 4.3. Comparison to Alternative Methods

In order to assess how the proposed method performs, a comparison is made between the results it achieves and those of two other methods reported in the literature: Liang et al. [16] and the method employed in the Kalibr calibration toolbox [20]. It should be noted that these estimate the transformation between a camera and an IMU, while the proposed method estimates the calibration between an IMU and its parent frame in the topological structure of the robotic system.

This has two implications. The first is that, in order to establish a comparison, a system containing both a camera and an IMU must be the subject of calibration. As such, the RiHIBot (see Figure 2) system is used for the calibration. The second is that the error terms used for the comparison must be those from the comparison to the ground truth of the camera–IMU transformation. For the proposed method, the estimated camera–IMU transformation is obtained using the transformations in the kinematic chain of the robot and the estimated Tsf:

(19) T^sc=Tfc·T^sf

where Tfc is the transformation, obtained through forward kinematics, between the camera sensor and the parent frame of the IMU.

Table 1 shows the errors, obtained through comparison of the estimated camera–IMU transformation to the ground truth for the RiHIBot system and for each of the three calibration methods. The table displays both the translation error, $E_{t}$, and the rotation error, $E_{r}$. It should be noted that the default configurations for each of the alternative methods were used for this comparison. Furthermore, the same dataset was used for the calibration with all three methods to ensure a fair comparison.

The proposed method achieved better results than both alternatives. For the translation, while the two methods used for comparison reached error values between 2 cm and 3 cm, the proposed method achieved an error value under 3 mm. Similarly, the rotation error achieved using the proposed method is under 0.01 degrees, while Kalibr [20] reported an error over 0.02 degrees, and Liang et al. [16] reported an error over 5 degrees.

### 4.4. Sensitivity to IMU Noise

As mentioned, in order to assess the applicability of this calibration method, we explore its sensitivity to noise in the IMU data. We collected calibration results for varying values of noise in both the accelerometer data and gyroscope data. These results can be found in Table 2.

The stratified-K-fold cross-validation method was employed to collect these values. For every noise value pair $\left(\lambda_{n,a},\lambda_{n,\omega }\right)$, three folds were used and for each fold, five different calibrations were carried out. Each calibration uses a different random seed for sampling $n_{a}$ and $n_{\omega }$. These five runs result in calibrations with five different errors for the various datapoints in the IMU data stream. As such, the results presented reflect both different sets of data used for calibration and different noise samplings along the IMU measurements.

The table shows there is a clear increase in the errors for RiHIBot when the noise in the accelerometer measurements has an amplitude of $0.001\;{\mathrm{ms}}^{-2}$. Both when the gyroscope noise values is null and when it varies with the accelerometer noise, there is a distinct increase in the translation error. Consider the case where there is no noise in the gyroscope measurements, i.e., $\lambda_{n,\omega }=0$. For values of $\lambda_{n,a}$ under $0.001\;{\mathrm{ms}}^{-2}$, final translation errors remain under 5 mm. For $\lambda_{n,a}\geq 0.001\;{\mathrm{ms}}^{-2}$, however, those same errors climb to over 5 mm, reaching as high as 16 mm for $\lambda_{n,a}=0.005\;{\mathrm{ms}}^{-2}$. For the rotation error, though, a significant increase can only be seen for $\lambda_{n,a}\geq 0.005\;{\mathrm{ms}}^{-2}$. Even so, all post-calibration rotation errors are under 0.02 degrees.

Varying the noise in the gyroscope seems to have no impact on the final translation and rotation errors for RiHIBot. For null values of accelerometer noise $\lambda_{n,a}$ and for all tested values of gyroscope noise $\lambda_{n,\omega }$, all final translation errors are under 4.3 mm. Similarly, all rotation errors for these calibrations are under 0.0066 degrees. Furthermore, for the calibrations where the accelerometer and gyroscope noise vary together, the results are almost equal to those achieved with varying accelerometer noise and null gyroscope noise.

For the case of BarBot, however, the same observations do not hold. For all tested noise values for both the accelerometer and gyroscope, translation errors remain under 3 mm. Though some increases can be seen in the translation errors for increasing values of accelerometer noise and when $\lambda_{n,\omega }=0$, these increases are relatively small, with a difference under 0.5 mm between calibrations using $\lambda_{n,a}=0\;{\mathrm{ms}}^{-2}$ and $\lambda_{n,a}=0.01\;{\mathrm{ms}}^{-2}$. Similar error values are also observed when the accelerometer noise and gyroscope noise values increase in tandem with each other.

A possible explanation for why this increase in error is seen in the case of RiHIBot and not in the case of BarBot is the presence of gravity in the first case and its absence in the second. This indicates that there might be errors originating in the compensation of gravity in the IMU acceleration measurements. One hypothesis that might explain this is that there may be a temporal misalignment between the transformation data used to compute Tsw and the IMU datapoints. Such a misalignment might lead to an improper compensation of gravity which in turn affects the comparison established between derived and measured IMU data. Further exploration of this hypothesis should be carried out in future works.

Nevertheless, these calibration results indicate that the proposed method is appropriate for application to industrial sensors. Consider the industrial IMU ADIS16495 (https://www.analog.com/en/products/adis16495.html?doc=ADIS16495.pdf (accessed on 5 January 2026)). The accelerometer for this sensor displays a typical noise density of 17 μg/$\sqrt{\mathrm{Hz}}$. At a bandwidth of 150 Hz, this translates to a Root Mean Square (RMS) error, for each accelerometer sample, of 0.0002 ${\mathrm{ms}}^{-2}$. For these error values, i.e., when $\lambda_{n,a}\leq$ 0.0002 ${\mathrm{ms}}^{-2}$, the proposed method displays translation errors under 4.5 mm and the rotation errors under 0.01 degrees.

### 4.5. Sensitivity to Initial Error

As mentioned, in order to study the sensitivity of this calibration method to the initial error, several calibrations were carried out with varying values of initial translation and rotation errors in the estimated transformation, T^sf.

Results were collected for an array of initial translation errors between 0 m and 1 m, and for initial rotation errors between 0 rad and 1 rad. In a similar fashion to the IMU noise sensitivity study in Section 4.4, calibrations were carried out with only translation errors present in T^sf, others with only rotation error, and calibrations with both translation and rotation error in the initial estimate. Once more in accordance with the experiments in Section 4.4, a stratified K-fold cross-validation was employed, with three folds. For each fold, five different calibrations were carried out using different random seeds. This ensures the error vectors added to both the translation and rotation have different directions for each calibration.

In the case of both the BarBot and RiHIBot systems, there is almost no variation in the final errors for different initial translation and rotation error values. In the case of both systems, the final translation errors remain under 3 mm with no significant variation across different initial error values. The same observation can be made about the rotation errors, which for BarBot remain under 0.05 degrees and under 0.007 degrees for the case of RiHIBot.

The lack of variation in the post-calibration errors indicates that the least squares optimization used to minimize the cost function established in Equation (13) is able to converge on an optimal solution. It might be worth noting that some of the initial errors used for testing the calibration are mostly theoretical and would rarely, if ever, appear in a practical application. As such, the results seem to indicate that, for any realistic initial errors in the estimated transformation, the optimization of the cost function is not susceptible to falling into local minima.

## 5. Conclusions

In this paper, a novel method for the extrinsic calibration of IMUs in robotic systems was presented. The proposed approach does not make use of IMU data integration and is not dependent on a second sensor with a rigid spatial relation to the inertial sensor. On the one hand, integration errors are avoided by taking the approach of deriving the world–IMU pose estimate and establishing a comparison between the derived data and the measured IMU data. On the other hand, framing the calibration as the estimation of the pose of the IMU in relation to its parent frame in the topological structure of the system allows the need for a second sensor to be bypassed. The independence of the presence of a second sensor with a fixed spatial transformation to the IMU, as well as the use of a derivative method instead of an integrative method, are the main advantages of the proposed method.

The method was tested using simulated robotic systems. Using these same systems, calibration results were compared to those of other methods in the literature. The proposed method displays lower post-calibration translation and rotation errors than the alternatives it was tested against. Experiments were conducted to investigate the sensitivity of the proposed approach to both noise in the IMU measurements and the initial error in the estimate of the calibrated transformation. The first of these experiments revealed that the method is robust when faced with accelerometer noise levels comparable to those of industrial-grade inertial units. The second of these experiments revealed that the optimization of the defined cost function reliably converges on an optimal solution for any realistic initial estimate of the pose of the IMU in relation to its parent frame.

Possible future avenues for research include the refining of the gravity compensation method employed and the inclusion of a temporal calibration process of the robotic system. A further study into possible noise mitigation methods may reveal broader applicability to lower-grade sensors. Additionally, future works should include experiments conducted using real robotic systems. A study should also be conducted on the merit of different optimization methods in the context of the proposed calibration method to assert which method is better suited in that context.

## Figures and Tables

**Table 1:** Translation and rotation errors of the calibration of the RiHIBot system using different methods.

Method	$E_{t}$ [mm]	Er [°]
Kalibr [20]	20.283	0.261
Liang et al. [16]	27.757	6.538
Proposed Method	2.720	0.0069

**Table 2:** Calibration results regarding BarBot and RiHIBot for varying levels of noise in both accelerometer and gyroscope data.

Accelerometer Noise [ms^−2^]	Gyroscope Noise [rad/s]	BarBot		RiHIBot	
		$E_{t}$ [mm]	$E_{r}$ [°]	$E_{t}$ [mm]	$E_{r}$ [°]
0	0	2.2807	0.0543	4.2955	0.0064
0.00001	0	2.2884	0.0405	4.2985	0.0064
0.0001	0	2.2905	0.0406	4.3118	0.0064
0.0002	0	2.2929	0.0406	4.3385	0.0063
0.0005	0	2.3004	0.0410	4.5130	0.0063
0.001	0	2.3143	0.0418	5.2027	0.0062
0.002	0	2.3462	0.0442	7.5546	0.0066
0.005	0	2.4729	0.0562	16.3861	0.0097
0.01	0	2.7657	0.0822	31.8585	0.0171
0	0.00001	2.2883	0.0405	4.2969	0.0064
0	0.0001	2.2878	0.0406	4.2975	0.0064
0	0.0002	2.2874	0.0407	4.2973	0.0064
0	0.0005	2.2864	0.0413	4.2965	0.0064
0	0.001	2.2845	0.0428	4.2959	0.0064
0	0.002	2.2809	0.0475	4.2955	0.0064
0	0.005	2.2701	0.0716	4.2928	0.0064
0	0.01	2.2525	0.1229	4.2891	0.0065
0.00001	0.00001	2.2884	0.0405	4.2983	0.0064
0.0001	0.0001	2.2902	0.0406	4.3115	0.0064
0.0002	0.0002	2.2926	0.0408	4.3383	0.0063
0.0005	0.0005	2.2995	0.0414	4.5129	0.0063
0.001	0.001	2.3125	0.0429	5.2017	0.0062
0.002	0.002	2.3427	0.0477	7.5507	0.0066
0.005	0.005	2.4645	0.0712	16.3719	0.0097
0.01	0.01	2.7498	0.1219	31.8297	0.0172

## Data Availability

The original contributions presented in this study are included in the article. Further inquiries can be directed to the corresponding author.
